# The neurosurgical outpatient clinic: comparison between accesses in public and private activities

**DOI:** 10.1186/s12913-024-10571-6

**Published:** 2024-01-25

**Authors:** Marta Menegatti, Nunzia Del Villano, Alba Scerrati, Francesco Travaglini, Luca Ricciardi, Giorgio Lofrese, Michele Alessandro Cavallo, Pasquale De Bonis

**Affiliations:** 1 Neurosurgery Sant’Anna University Hospital of Ferrara, Ferrara, Italy; 2 Department of Translational Medicine University of Ferrara, Ferrara, Italy; 3 NESMOS Department Sapienza University of Rome, Rome, Italy; 4 Department of Neurosciences, Neurosurgery Division “M Bufalini” Hospital Cesena, Cesena, Italy

**Keywords:** Neurosurgery, Outpatient clinic, Private clinic, Public hospital, Healthcare resources

## Abstract

**Background:**

Neurosurgical clinic assesses presence and extent of pathologies of central and peripheral nervous system or disorders affecting the spine, to identify most effective treatment and possible recourse to surgery. The aim of the study is to evaluate the appropriateness of request for a neurosurgical consult both in private and in public outpatient clinics.

**Materials and methods:**

We collected and analyzed all the reports of outpatient visits of public and private clinic over a period between January and December 2018.

**Results:**

There were 0.62% real urgent visits in the public sector and 1.19% in the private sector (*p* = 0.05). Peripheral pathologies represented 12.53% and 6.21% of pathologies evaluated in public and private sector respectively (*p* < 0.00001). In addition, 15.76% of visits in public lead to surgery, while they represented 11.45% in private (*p* = 0.0003).

**Conclusions:**

No study is available comparing accesses of patients in neurosurgical outpatient clinics. In public clinic, visits are booked as urgent on the prescription of the general practitioner: in reality, only 5% of these visits were really confirmed as urgent by the specialist. Peripheral pathologies are more frequent in public clinic, while cranial pathologies are more frequent in private one. Patients with cranial pathologies prefer to choose their surgeon by accessing private clinic.

## Introduction

The neurosurgical clinic represents a specialized service in which the neurosurgeon carries out a visit aimed at assessing the presence and extent of pathologies of the central and peripheral nervous system or disorders affecting the spine, in order to identify the most effective treatment and the possible recourse to surgery. The visit, in addition to an accurate diagnosis, in the provision of an intervention allows the planning of the times and methods of the intervention itself.

In many healthcare systems, the general practitioner (GP) plays a pivotal role as a gatekeeper, controlling patient access to secondary and tertiary care based on the principle of necessity. In the current Italian context, GPs do not have formal guidelines when referring to a neurosurgical opinion. Usually, they only have a limited range of diagnostic images available, yet they have the means to refer patients directly to allied healthcare professionals.

Specialist ambulatory services, including visits and diagnostic activities, are provided either by the ASLs (local sanitary holdings) or by accredited public and private facilities, with which ASLs have agreements. Services are listed in specific formularies that vary from region to region. People can access specialized care either on the recommendation of their GP or, for some services, by booking an appointment directly through a central booking point [single booking center (CUP)] [1]. Another alternative is to contact a clinic or private doctor directly.

Like almost all branches of specialist medicine, the same service can therefore be provided by specialists who work in the public sector or by those who work in the private sector. In the first case, the patient can take advantage of this service referred by GPs and by reservation at the CUP. In the second case, the patient can go directly to the specialist without any notification from the family doctor, paying a higher price. In both cases it was found that, in the face of an increasing number of patients who access the outpatient neurosurgery service, only a modest percentage of clinical pictures are indicated for surgery. This means that most access is inappropriate, leading to inefficient use of healthcare resources. The aim of the study is to evaluate the appropriateness of request for a neurosurgical consult both in private and in public outpatient clinics.

## Materials and methods

The neurosurgical service of the Sant’Anna University Hospital, one of the 5 Departments of Neurosurgery of the Emilia Romagna Region, based in Ferrara, serves a population of about 350,000 people living in the North East of Italy. The service is currently run by six full-time neurosurgeons. Patients are referred by GPs or hospital specialists directly to our emergency service or outpatient clinics. All referrals are evaluated by a consulting neurosurgeon. All the reports of outpatient visits of the neurosurgery clinic and of private visits were analyzed over a period of time between January and December 2018 (before COVID-19 pandemic).

Patient visits were recorded on a spreadsheet using Excel 2018.

All the specialist referrals to the Sant’Anna neurosurgery outpatient service were also analyzed with the same outcome measures. Patients referred by the emergency departments were not included in the study. Patients who were acutely referred to our services by specialist teams were also excluded from the study, as were patients who did not show up for outpatient appointments.

During each visit, the following patient data were reported: anonymous code associated to the patient, date of birth, age and date of the visit. Each patient was asked the type of pathology for which he was referred to the clinic, which was subsequently classified as cranial, spinal or peripheral disease. Once the disease was investigated, it was assessed as urgent (i.e. requiring treatment within a few days) or non-urgent. When recent radiological documentation was not available, the evaluation was based only on the anamnesis and on the physical examination. Only patients with clinical suspect of neurosurgical disease amenable for surgical treatment were asked further radiological investigations. For each diagnosis made, the neurosurgeon assessed whether there was a surgical indication, explaining risks and benefits to the patient. The patient could immediately accept the surgery, refuse, or could take time to reflect and communicate the decision later. Another aspect of the examination evaluated is the congruence of the pathology, i.e. whether the type of pathology or symptomatology does not fall within the sphere of neurosurgery, but of another specialty or whether it may be patients who have made an appointment some time before but in the meantime the symptomatology has been resolved or, again, booking errors.

In any case, the neurosurgeon gave instructions for therapy or follow-up. Hence, we also considered any form of therapeutic intervention. The key data on the outcomes sought were the number of visits actually requested, the number of neurosurgical operations performed out of the total number of visits and the number of operations refused.

The statistical method used for data analysis is the exact chi-squared and Fisher’s test and the student’s t-test. A *p*-value < 0.05 was defined as statistically significant.

## Results

There were 3150 accesses to the public clinic and 1302 accesses to the private clinic in the period between January and December 2018.

In the public clinic, females amounted to 1689 (53,52%) while males to 1461 (46,48%), and in the private clinic 713 females (54,76%) and 589 males (45,24%) were recorded. No differences between males and females in the two clinics (*p* = 0.49) were detected, as shown in Table 1.

**Table 1:** Sex of patients

Clinic	Female	Male	Total
**Public**	53,52% (1689)	46,48% (1461)	3150
**Private**	54,76%	45,24%	1302
**Chi-square**		0,48	
**P**		**0,4865**	
**Chi-square corrected**			
**Yates**		0,44	
**P**		0,5054	
**Odds-ratio**		0,96	

As regards to age, patients in both clinics were classified into two categories: younger or older than 65. Data from the public clinic show that patients under 65 years of age were 1887 (59.90%), while patients over 65 years of age were 1263 (40.10%). Data from the private clinic indicate that patients under 65 years of age were 774 (59.45%), while patients over 65 years of age were 528 (40.55%). Again, no statistically significant difference (*p* = 0.79) was found (Table 2).

**Table 2:** Age of patients

Clinic	Over 65	Under 65	Total
**Public**	40,10% (1263)	59,90% (1887)	3150
**Private**	40,55% (528)	59,45% (774)	1302
**Chi-square**		0,08	
**P**		**0,7769**	
**Chi-square corrected**			
**Yates**		0,06	
**P**		0,8028	
**Odds-ratio**		1,02	

**Fig. 1:**
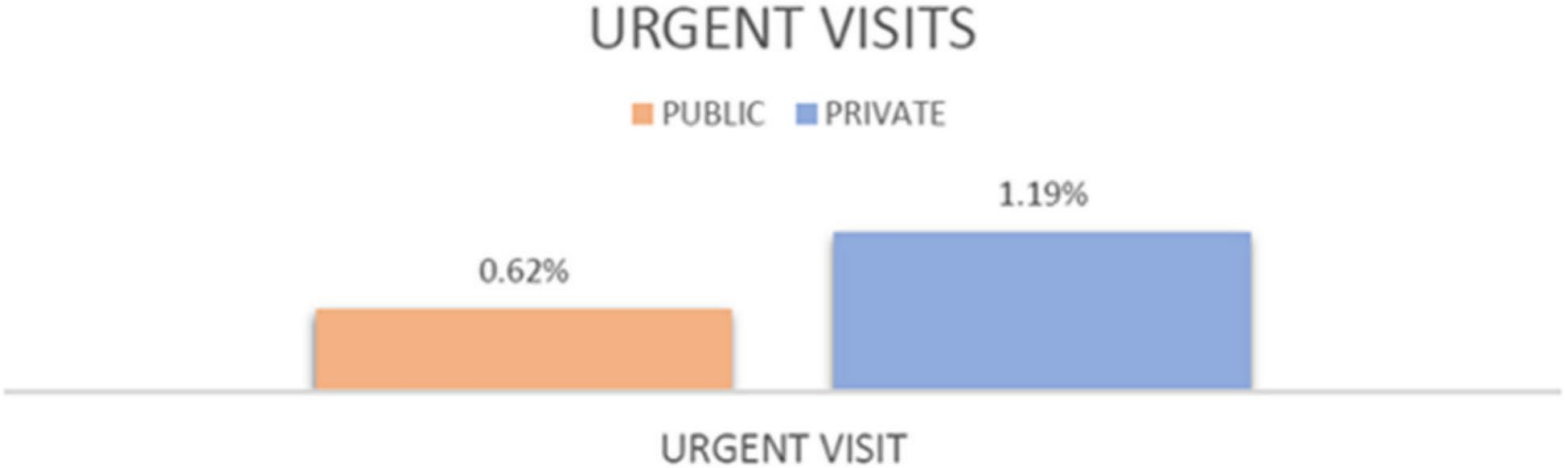
Urgent Visits

The general practitioner includes in the request for the first visit whether it is urgent or not. The number of visits booked as urgent at the public clinic was 378 (12%), but only 19 (5.03%) of these were really urgent. For private neurosurgery visits, since communication between the patient and the neurosurgeon is direct, the percentage of really urgent visits out of the total number of visits was compared. The data show that there were 19 out of 3072 (0.62%) real urgent visits in the public sector and 15 out of 1257 (1.19%) in the private sector. The difference in urgent visits was statistically significant (*p* = 0.05), as can be seen in Table 3 and Fig. 1.

**Table 3:** Urgent visits

Clinic	Urgent	Non urgent	Total
**Public**	0,62% (19)	99,38% (3053)	3072
**Private**	1,19% (15)	98,81% (1242)	1257
**Chi-square**		3,78	
**P**		**0,0518**	
**Chi-square corrected**			
**Yates**		3,08	
**P**		0,0792	
**Odds ratio**		0,52

Incongruous pathologies represented 2% in both sectors, 63 out of 3150 visits in the public clinic and 26 out of 1302 visits in the private clinic. No statistically significant difference (*p* = 0.99) was found (Table 4).

**Table 4:** Congruity of visits

Clinic	Congruous	Incongruous	Total
**Public**	98,00% (3087)	2,00% (63)	3150
**Private**	98,00% (1276)	2,00% (26)	1302
**Chi-square**		0,00	
**P**		**0,9947**	
**Chi-square corrected**			
**Yates**		0,01	
**P**		0,9116	
**Odds-ratio**		1,00	

**Fig. 2:**
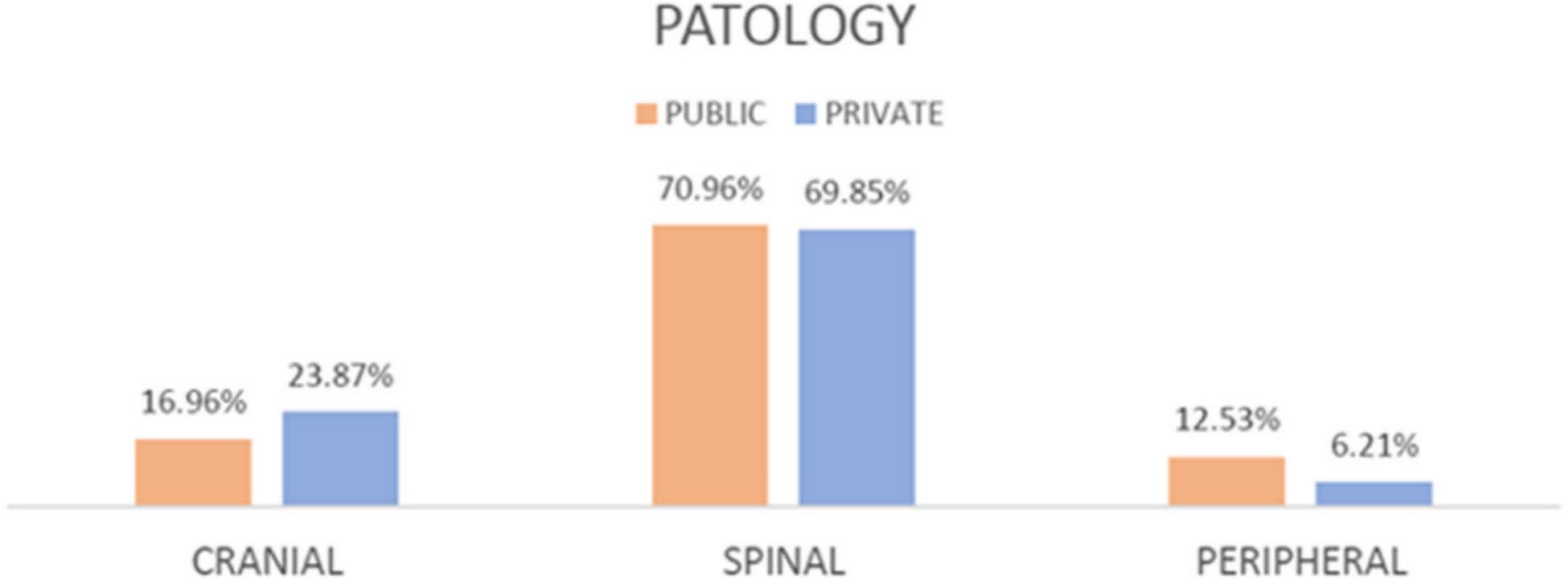
Pathologies are classified in three macro areas: cranial, spinal, peripheral

**Fig. 3:**
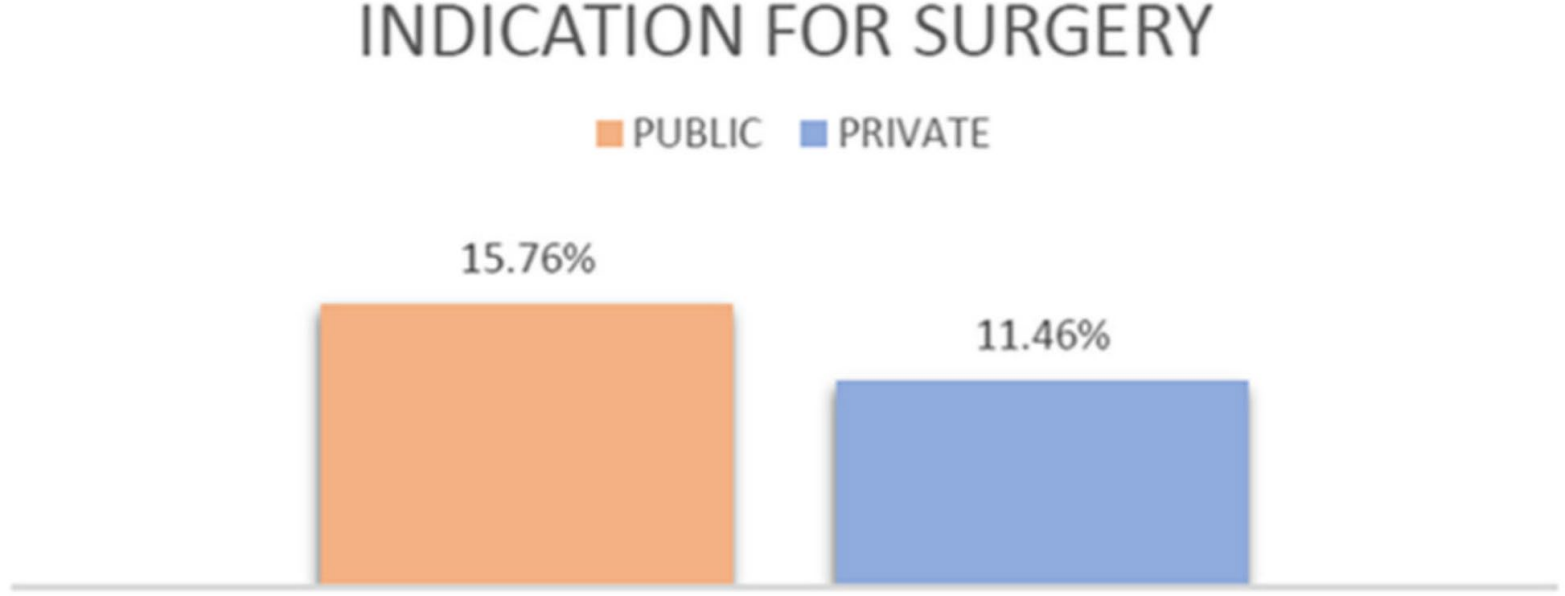
Percentage of pathologies with indication for surgery

In public visits, 521 out of 3072 (16.96%) were cranial pathologies while 300 out of 1257 (23.87%) were in private clinics. Spinal pathologies, on the other hand, amounted to 2180 out of 3072 (70.96%) in the public clinic, while 878 out of 1257 (69.85%) for private. Peripheral pathologies represented 12.53% (385 out of 3072) of all pathologies evaluated in the public clinic and 6.21% of pathologies evaluated in the private sector (78 out of 1.557). The statistical analysis performed by the Fisher exact test showed a statistically significant difference (*p* <  0.00001) (Table 5, Fig. 2). 15.76% of the total number of visits (484 out of 3072) in public lead to surgery, while they represented 11.46% (144 out of 1257) in private. This difference was statistically significant (*p* = 0.0003) (Table 6, Fig. 3).

**Table 5:** Pathologies are classified in three macro areas: cranial, spinal, peripheral

Clinic	Cranial	Spinal	Peripheral
**Public**	16,96% (521)	70,96% (2180)	12,53% (385)
**Private**	23,87% (300)	69,85% (878)	6,21% (78)
**Chi-square**		56,08	
**P**		**< 0,00001**

**Fig. 4:**
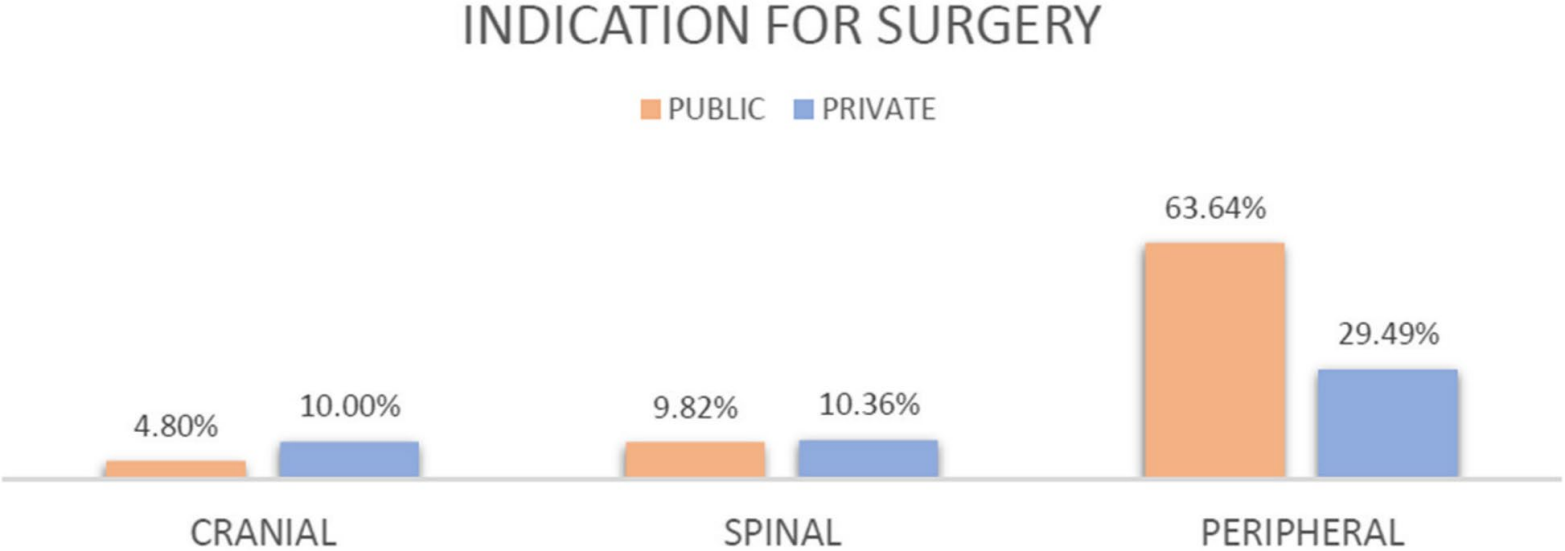
Percentage of indication for surgery according to the type of pathology

Cranial pathologies with surgical indications represented 4.80% (25 out of 521) in public and 10% (30 out of 300) (*p* = 0.0041) in private. The spinal pathologies with surgical indications in the public clinic were 9.82% (214 out of 2180) while in the private they were 10.36% (91 out of 878). This difference was not statistically significant (*p* = 0.6473). The peripheral nerve pathologies with surgical indication in the public clinic, amounted to 63.64% (245 out of 385) while in the private clinic they were 29.49% (23 out of 78). This difference was statistically significant (*p* < 0.00001) (Table 7*,* Fig. 4).

**Table 6:** Percentage of pathologies with indication for surgery

Clinic	Surgical	Non surgical	Total
**Public**	15,76% (484)	84,24% (2588)	3072
**Private**	11,46% (144)	88,54% (1113)	1257
**Chi-square**		13,23	
**P**		**0,0003**	
**Chi-square corrected**			
**Yates**		12,88	
**P**		0,0003	
**Odds ratio**		1,44

**Table 7:** Percentage of indication for surgery according to the type of pathology

Clinic	Cranial	Spinal	Peripheral	Indication for surgery
**Public**	4,80% (25)	9,82% (214)	63,64% (245)	484
**Private**	10,00% (30)	10,36% (91)	29,49% (23)	144
**Chi-square**	8,24	0,21	31,03	
**P**	**0,0041**	**0,6473**	**0,00001**	
**Chi-square**				
**Corrected yates**	7,43	0,15	29,64	
**P**	0,0064	0,6959	0,0000	
**Odds-ratio**	0,45	0,94	4,18

**Fig. 5:**
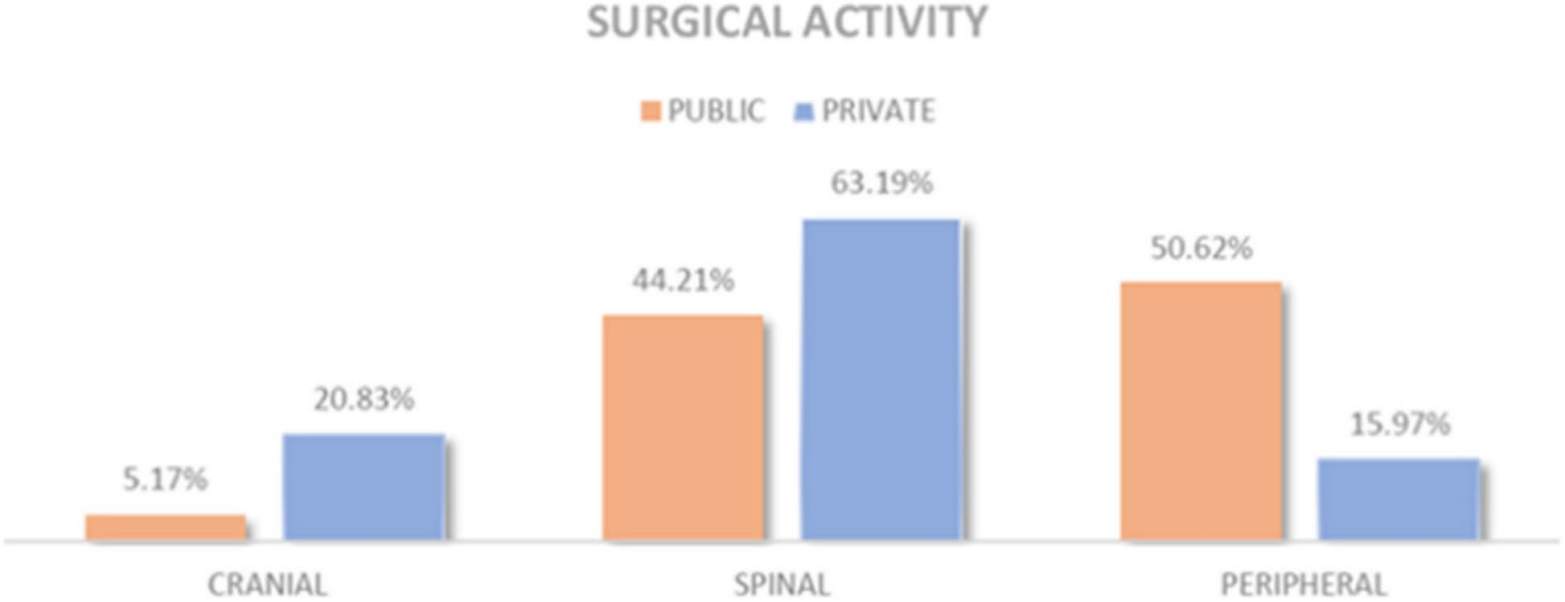
Surgical activity according to type of pathology

For patients referred by outpatient clinic, cranial surgery in the public sector accounted for 5.17% (25 out of 484) of total operations, while in the private sector it accounted for 20.83% (30 out of 144). Spinal surgery represented 44.21% (214 out of 484) of surgeries in the patients evaluated in public hospital, while 63.19% (91 out of 144) in the private hospital. Finally, peripheral surgery represented 50.62% (245 out of 484) of surgeries, while in the private sector it represented 15.97% (23 out of 144). There was a significant difference between the two sectors, public and private (*p* = 0.00001) (Table 8*,* Fig. 5).

**Table 8:** Surgical activity according to type of pathology

Clinic	Cranial	Spinal	Peripheral	Total
**Public**	5,17% (25)	44,21% (214)	50,62% (245)	484
**Private**	20,83% (30)	63,19% (91)	15,97% (23)	144
**Chi-square**		70,56		
**P**		**0,00001**

The waiting time for surgery following a public neurosurgical examination was 63.28 days (standard deviation 73.05), while 51.36 days (standard deviation 65.63) for private. The difference was not significant (student t test *p* = 0.0792). The number of refused surgery was 22.73% in the public outpatient visits (110 out of 484) and 13.89% in the private outpatient visits (20 out of 144). The difference was statistically significant (*p* = 0.0216) (Table 9).

**Table 9:** Percentage of refused interventions

Clinic	Refused	Accepted	Total
**Public**	22,73% (110)	77,27% (374)	484
**Private**	13,89% (20)	86,11% (124)	144
**Chi-square**		5,28	
**P**		**0,0216**	
**Chi-square corrected**			
**Yates**		4,76	
**P**		0,0292	
**Odds ratio**		1,82	

**Fig. 6:**
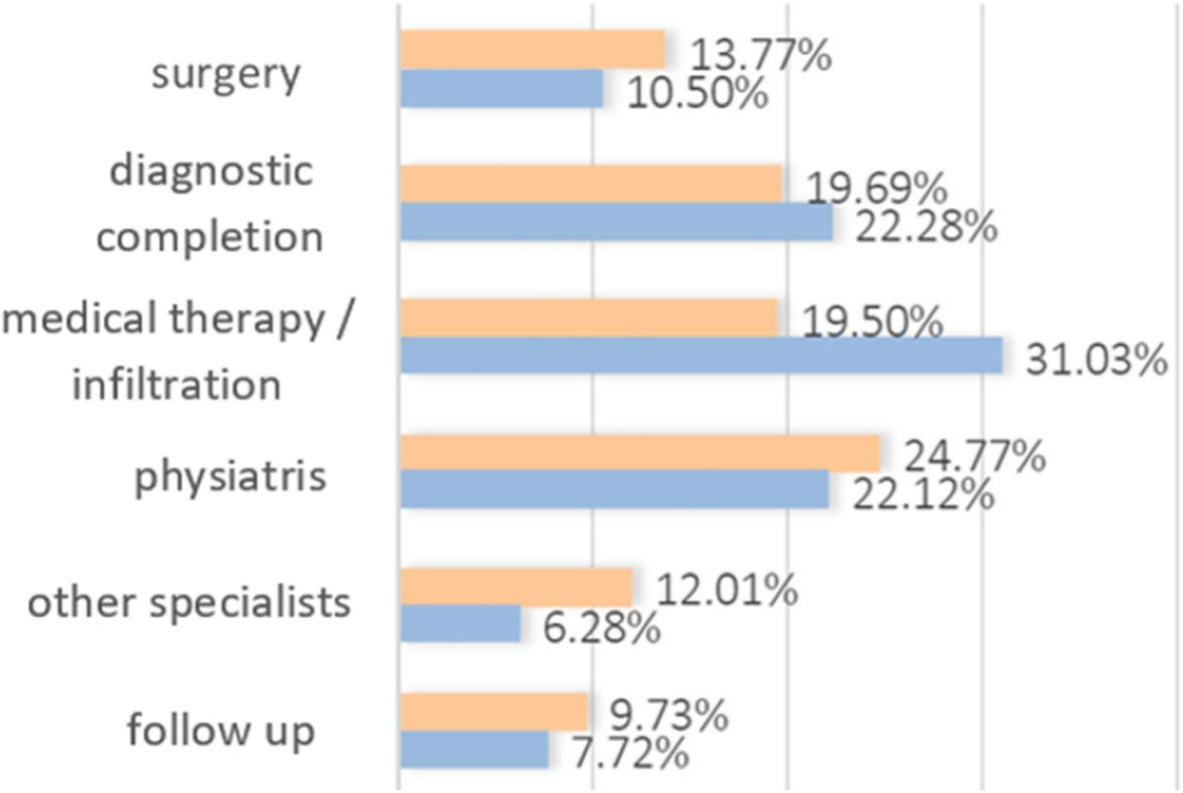
Distribution of therapeutic alternatives different from intervention

Finally, surgery was recommended in 13.77% of cases (423 out of 3072) in the public outpatient clinic and in 10.50% (132 out of 1257) in the private one. Diagnostic completion was prescribed in 19.69% (605 out of 3072) in the public and 22.28% (280 out of 1257) in the private. Medical therapy or infiltration based on corticosteroids was prescribed in 19.5% (599 out of 3072) in public and in 31.03% (390 out of 1257) in private. The physiatrist assessment was recommended in 24.77% of cases (761 out of 3072) in public and in 22.12% (278 out of 1257) in private. Follow up was recommended in 9.73% in public and in 7.72% in private (Fig. 6).

## Discussion

Aim of our study was to evaluate the pertinence of requested outpatient clinic neurosurgical evaluations in a public or private context.

We decided to collect data about this topic due to the increasing evidence of studies reporting the referral and consultation communication between primary care and specialist physicians and the appropriateness of specialist in outpatient clinics [2–5].

For “appropriate patient” we could consider a patient sent for evaluation by the GP or other specialists and suffering from a kind of disease in which the specific surgery of the requested consult is indicated. In other countries similar studies have been performed. For example Kamat et al. [3] showed that specialist referrals result in a proportionally greater number of therapeutic surgical interventions than GP referrals. They concluded that the development of relevant guidelines for primary care referral to a neurosurgical service, could facilitate initiation of appropriate investigations in primary care.

Our results showed there are no major differences with regard to the public and private clinics activities. On the other hand, it emerged that there was a slightly higher percentage of urgent visits in the private clinic (1.19% vs 0.62%, *p* = 0.0518). This could be explained by the fact that generally the waiting list for a public visit is longer than the private one. Several studies evaluated this aspect. A study by the University of Edinburgh in 2013 analyzed separately the utilization of general practitioner and outpatient specialist services, estimating how some selected characteristics of the public health care system are related to the utilization of private outpatient care. Two basic mechanisms can influence the demand for private care. First, the disutility of private care utilization can be lower than the one of public care, due to shorter waiting times or higher quality of services [6]. However, it should be noted that the problem of waiting lists varies profoundly not only between European countries but also between individual Italian regions. In fact, in our hospital, the waiting time for an urgent visit to the public clinic is less than 7 days, while the average waiting time for a regular visit is 20 days. In the private clinic, the average waiting time is 8 days.

Our study showed that there are no age differences justifying the preferential use of a public or private clinic.

Our data also showed patients preferred private clinics for cranial pathologies. Indication for surgery for spinal pathologies did not differ in public or private (respectively 9.82% vs 10.36%). Indeed, a difference resulted in patients finally accepting the surgery 44.21% in public versus 63.19% in private. These data reflected the number of rejected interventions, 22.73% in the public versus 13.80% in the private. Probably, patients accessing private clinics are more prone to accept therapy proposed by the surgeon, including surgery. This could be explained by the fact that patients who choose to perform a private visit choose the surgeon, and they may feel more confident believing that the therapeutic effort is better focused on them. Factors affecting patient satisfaction following a public or private visit have been investigated in several studies such as a 2011 study by the Liverpool School of Tropical Medicine [7]. A systematic review of comparative studies was carried out that compared the quality of private outpatient health care compared to public in low and middle income countries [7]. The results have shown that, overall, the private sector performed better in relation to drug supply, responsiveness, and effort. No difference between provider groups was detected for patient satisfaction or competence. Synthesis of qualitative components indicates the private sector is more client centered [7].

Another study showed 5 factors as significant predictors of overall patient satisfaction: physician personal modality, confidence interval, time spent with the physician, time to get an appointment and explanation of what was done [8]. In contrast, there was no statistically significant association between the overall satisfaction scores and the following factors: length of wait at the clinic, reaching the clinic by telephone, convenience of the location of the clinics and the physician’s technical skills [8].

Most of the visits were required for spinal diseases, as these represented 70% in both the public and private sectors. However spinal diseases for which surgery was indicated were only 9.82% in the public and 10.36% in the private. Probably, before GPs referred patients for neurosurgical consults, they should consider other therapies, such as physical medicine evaluation, infiltrative corticosteroid therapy or evaluation by another specialist. The fact that this does not happen may be due to the lack of guidelines to help general practitioners refer patients with spinal diseases to the neurosurgeon, as also confirmed by a study by Kamat et al. [3] These authors also showed neurosurgeons spend most of their time screening patients rather than operating on them and that an increased number of outpatient clinics did not result in an increased number of surgeries [3]. Therefore, the development of relevant guidelines for the referral of primary care to a neurosurgery service appears justified and could facilitate the initiation of appropriate investigations in primary care. Avoiding inappropriate referrals could reduce waiting times for both surgical consultation and lumbar spine surgery for those patients who require it.

The creation of precise guidelines aimed at helping GPs correctly direct patients to the most appropriate appointments could reduce waiting times for visits, enable patients in real need to access the right outpatient services more quickly, and consequently increase patient satisfaction. Dasic et al. conducted a study with the purpose of identifying protocols, guidelines, and best practices for the management of traumatic brain injuries (TBI), aiming to provide the best possible care for patients. They concluded that indeed, adherence to guidelines, the development of best practices, and the use of standard operating procedures (SOP) promote standardization of practice and streamline both emergency and elective activities. Furthermore, they also highlighted how the transition to virtual consultations and the use of telemedicine, to expedite outpatient clinical appointments, in their case, for follow-ups, can improve patient satisfaction and adherence to prescribed treatments and recommendations [9].

Indeed, we reported data from a restricted reality, different from other realities in our same country and even more from other countries abroad.

However, summarizing the final results, they seem to suggest a huge waste of resources in terms of appropriateness of the request for a neurosurgical outpatient clinic consult, both in private or in public. The waste of resources is reflected in economic (for public health system or the patient himself), time (of the specialist and the patient) and professionality.

In order to improve the appropriateness of the referrals we can suggest the following initiatives:

1. Facilitation of the relationship between GPs and the specialists of the area with the possibility to a direct and immediate contact in case of need.
2. Yearly planned update joint workshops in which they could discuss main topic and indications for patients referral to the outpatient clinic.
3. Clear scientific society guidelines about specific disease that could help GPs to refer or not the patient to the specialists.

These initiatives could be applied in any country and independently from a public or private health system.

### Limitations

A limitation of this study is the fact that it only took patients from one hospital as a sample. Probably by expanding the study and therefore the casuistry, also involving other hospitals in different regions of the country, we would have obtained more significant statistically results. Another limitation of this study could be that linked to the situation of the Ferrara Hospital in which the timing for patient access to the public and private outpatient clinics are not so different.

## Conclusions

In the public clinic, the visits are booked as urgent on the prescription of the general practitioner, who first assesses the urgency: in reality, only 5% of these visits were really confirmed as urgent by the specialist. Peripheral pathologies are more frequent in the public clinic, while cranial pathologies are more frequent in the private one: patients with cranial pathologies prefer to choose their surgeon by accessing the private clinic.

Finally, waiting times for the clinic are on average 8 days for the private clinic and 7 days (urgent visits) or 20 days (ordinary visits) for the public clinic. There are no differences in the timing of the surgical waiting lists. So even in public health there are no longer waiting lists, patients who ask for a private visit do so by choice, not because there are solid reasons for inefficiency. Finally, it is essential to define guidelines that are useful for general practitioners to direct their patients to specialist examinations similar to those related to symptoms. In this way, inappropriate services would be reduced, consequently reducing complex waiting times and also the costs for the national health system.

## Data Availability

All data generated or analysed during this study are included in this article. The data can be made available upon reasonable request from the Corresponding author.
